# The neural dynamics of deficient memory control in heavily traumatized refugees

**DOI:** 10.1038/s41598-018-31400-x

**Published:** 2018-09-03

**Authors:** Gerd T. Waldhauser, Martin J. Dahl, Martina Ruf-Leuschner, Veronika Müller-Bamouh, Maggie Schauer, Nikolai Axmacher, Thomas Elbert, Simon Hanslmayr

**Affiliations:** 1 Department of Neuropsychology, Institute of Cognitive Neuroscience, Faculty of Psychology, Ruhr University Bochum, 44801 Bochum, Germany; 2 Max Planck Institute for Human Development, 14195 Berlin, Germany; 3 Department of Psychology, University of Konstanz, 78457 Konstanz, Germany; 4 School of Psychology, University of Birmingham, Edgbaston, B15 2TT Birmingham, United Kingdom

## Abstract

Victims of war, torture and natural catastrophes are prone to develop posttraumatic stress disorder (PTSD). These individuals experience the recurrent, involuntary intrusion of traumatic memories. What neurocognitive mechanisms are driving this memory disorder? Here we show that PTSD symptoms in heavily traumatized refugees are related to deficits in the effective control of memory retrieval. In a think/no-think task, PTSD patients were unable to forget memories that they had previously tried to suppress when compared to control participants with the same trauma history but without PTSD. Deficits in voluntary forgetting were clinically relevant since they correlated with memory intrusions in everyday life. Magnetoencephalography (MEG) recorded during suppression attempts revealed that PTSD patients were unable to downregulate signatures of sensory long-term memory traces in the gamma frequency band (70–120 Hz). Thus, our data suggest that the inability to suppress unwanted memories through modulation of gamma activity is related to PTSD symptom severity.

## Introduction

The world currently faces large migration movements from regions struck by armed conflict, poverty and failure of economic development^1^. Due to repeated life-threatening experiences, more than half of all refugees present with symptoms of mental disorders, and a substantial proportion keep suffering from trauma-related disorders such as PTSD, with limited psychological functioning^2^. Gaining a deeper understanding of the impact of traumatic stress on the human mind and brain and developing new therapeutic strategies is not only of medical but also of humanitarian and socioeconomic relevance.

A hallmark symptom of PTSD is the uncontrollable intrusion of memories of the traumatic event(s) that result in high levels of emotional arousal and distress^3^. During extreme occurrences of such unbidden reminiscences in flashbacks or nightmares these symptoms become so intense that the present is perceived as an illusion while the past is considered as the actual reality. What neurocognitive mechanisms are driving this memory disorder? In healthy participants, repeated, voluntary suppression of retrieving previously learned material leads to subsequent forgetting^4,5^. Several studies have proposed that the ability to voluntarily suppress unwanted memories could protect against the development of PTSD and alleviate the accompanying symptoms of memory intrusions^6^. Indeed, recent behavioral data suggest a link between deficits in memory suppression and PTSD^5^.

In the present study, we were able to uncover the neural mechanisms of deficient memory suppression in heavily traumatized refugees while controlling for the influence of potentially confounding factors. To this end, we compared participants with PTSD with control participants that were matched in terms of traumatic experiences, levels of depression and demographic factors in their performance in a think/ no-think (T/NT) task^4^. In addition, we measured magnetoencephalography (MEG), a brain imaging method with very high temporal and acceptable spatial resolution. We focused on MEG activity in the gamma frequency band as a neural marker of sensory long-term memory traces^7,8^. Our data show that PTSD patients were unable to downregulate sensory memory traces through repeated suppression attempts. In contrast to control participants, PTSD patients showed an initial increase and a subsequent high level of gamma activity during suppression and thereby failed to forget unwanted memories. This corresponds to the patients’ futile attempts to control their traumatic memories in everyday life^3^.

## Results

### Sample description

Twenty-four refugees (5 female) from diverse European, African and Asian countries completed the study procedure. Based on the results of an extensive structured clinical interview, participants were divided into two subgroups with eleven (two female) participants fulfilling DSM-IV^9^ criteria for PTSD. The Control group did not differ from PTSD patients in terms of demographic factors (See Table 1). Importantly, Controls and PTSD patients reported that they had experienced a similar number and diversity of traumatic events (‘trauma load’^10^). Based on the Beck Depression Inventory (BDI-II)^11,12^, a similar number of participants in each group showed clinically relevant symptoms of depression. According to the assignment, the frequency of re-experiencing traumatic memories as assessed by means of the Posttraumatic Diagnostic Scale (PDS; scale B)^13,14^ separated the two groups. Furthermore, PTSD patients as opposed to Controls reported to unsuccessfully suppress unwanted thoughts in everyday life as indicated by higher scores on the White Bear Suppression Inventory (WBSI)^15,16^.

**Table 1:** Differences between Control and PTSD Groups in Demographic and Clinical Variables, and Basic Memory Performance.

	Control	PTSD	Test statistic
*N*	13	11	
Sex (female)	3	2	*OR* = 1.350, *P* = 0.769 ϕ = 0.060, 95% CI = [0.18, 10.01]
Age (years)	20.83 (6.58)	23.41 (7.50)	*U* = 67.5, *P* = 0.817 *d* = 0.095, 95% CI = [−5.50, 2.75]
Trauma load (no. of different traumatic event types)	4.00 (4.00)	6.00 (4.00)	*U* = 46.5, *P* = 0.141 *d* = 0.619, 95% CI = [−3.00, 0.00]
PDS symptom severity (sum score)	6.00 (7.50)	23.00 (20.00)	*U* = 6.5, *P* < 0.001* d = 2.404, 95% CI = [−29.00, −10.00]
PDS subscale B	1.00 (2.50)	6.00 (10.00)	*U* = 15.0, *P* = 0.001* d = 1.796, 95% CI = [−10.00, −2.00]
WBSI	50.00 (23.00)	62.00 (17.00)	*U* = 29.0, *P* = 0.014* d = 1.163, 95% CI = [−23.60, −4.20]
BDI-II cutoff ≥ 17	7	9	*OR* = 0.259, *P* = 0.159 ϕ = 0.296, 95% CI = [0.04, 1.70]
Learning rate (%)	83.19 (6.91)	79.80 (6.06)	*t*(22) = 1.268, *P* = 0.218 *d* = 0.519, 95% CI = [−2.16, 8.94]
False alarms (%)	5.29 (4.78)	14.58 (13.73)	*t*(13)^†^ = 2.139, *P* = 0.054 *d* = 0.939, 95% CI = [−18.76, 0.168]

Number of observed cases or *M* (*SD*) for normally distributed and *Mdn* (interquartile range) for non-normally distributed variables. *Significant result (*P* < 0.05); ^†^Corrected for unequal variances as indicated by significant Levene’s Test. Confidence intervals are given for the difference of means (*t*-tests) or medians (Mann-Whitney *U*-tests) together with Cohen’s *d*, and for odd’s ratio (*OR*) together with ϕ-coefficients.

### Behavioral results

**Figure 1:**
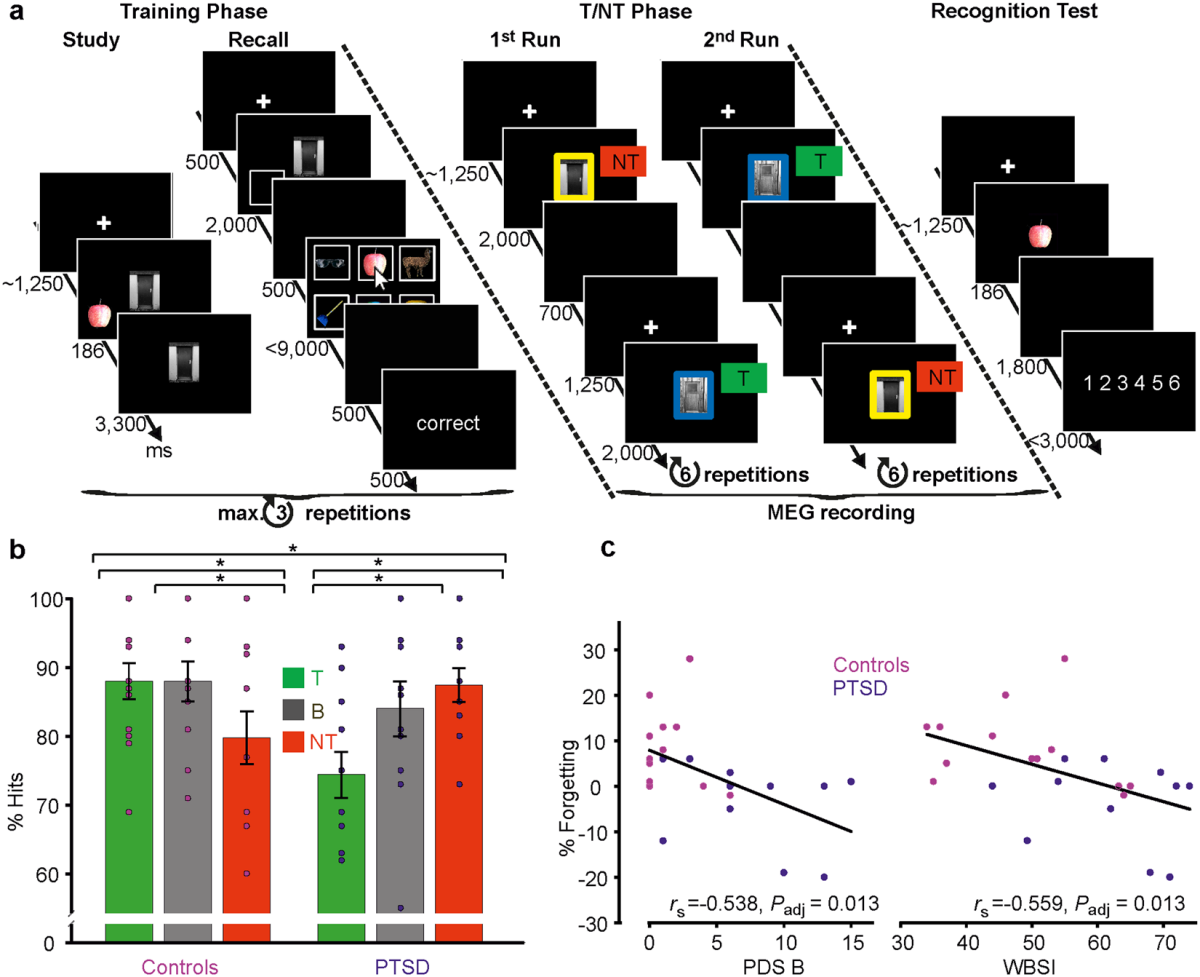
Experimental procedure and behavioral results. The T/NT task (**a**) consisted of three phases: Training, T/NT and recognition test phase. In the think condition (‘T’), participants were instructed to practice retrieval of everyday objects when being presented with a visual cue associated to the object during the training phase. In the no-think condition (‘NT’), participants were instructed to directly suppress the associated target object and to avoid any thought about it, without retrieving or focusing on alternative memories or thoughts. Memory performance in a subsequent surprise recognition test for T and NT items was compared to recognition performance for baseline (‘B’) items that were initially studied, but did not occur in the T/NT phase. (**b**) Item recognition memory performance showing a significant (P < 0.05) Group × Condition interaction and significant post-hoc comparisons as indicated by asterisks. Bars represent mean hit rates together with ±1 standard error of the mean and individual data points. See main text for statistical details. (**c**) Significant Spearman-rank correlations between suppression-induced forgetting and clinical measures of re-experiencing symptoms (PDS B) and thought suppression in everyday life (WBSI).

After the interview, participants completed a think/no-think task (Fig. 1a). In the think condition (‘T’), participants were instructed to practice retrieval of previously learned everyday objects when being presented with an associated visually complex cue. In the no-think condition (‘NT’), participants were instructed to directly suppress the associated target object and to avoid any thought about it, without retrieving or focusing on alternative memories or thoughts^17^. In contrast to substituting the to-be-suppressed target item with alternative mental content, this direct suppression strategy has been shown to induce inhibition of the retrieval process. Direct suppression leads to increased event-related potentials related to rapid inhibitory control and to reduction of late parietal potentials associated with conscious recollection^17^ as well as to a downregulation of hippocampal activity in fMRI studies^18^. In a subsequent surprise recognition test, memory performance for T and NT items was compared to recognition performance for baseline (‘B’) items that were initially studied, but did not occur in the T/NT phase.

In addition to a main effect of Condition (T, NT, B; *F*(2, 44) = 3.497, *P* = 0.039, η_*p*_^2^ = 0.137), memory performance in the three conditions differed between Controls and PTSD patients as indicated by a significant interaction analysis (*F*(2, 44) = 17.039, *P* < 0.001, η_*p*_^2^ = 0.436; see Fig. 1b). There was no main effect of Group that would indicate a general performance difference between PTSD patients and Controls (*F*(1, 22) = 0.638, *P* = 0.433, η_*p*_^2^ = 0.028). In line with numerous studies^19,20^, controls were able to intentionally suppress unwanted memories as indicated by a significant decrease in hit rates for NT items when compared to B items (*t*(12) = 3.499, *P* = 0.004, *d* = 0.623, 95% CI = [−13.45, −3.13]) and T items (*t*(12) = 4.529, *P* = 0.001, *d* = 0.518; 95% CI = [−12.21, −4.28]). In contrast, PTSD patients showed no difference between NT and B items (*t*(10) = 1.240, *P* = 0.243, *d* = 0.271, 95% CI = [−2.75, 9.66]). However, there was a significant decrease in hit rates for T when compared to B items (*t*(10) = 2.419, *P* = 0.036, d = 0.779, 95% CI = [−18.53, −0.76]) and NT (*t*(10) = 5.379, *P* < 0.001, d = 1.275, 95% CI = [−18.53, −7.68]). Thus, a significant difference in both, baseline-corrected suppression-induced forgetting (B-NT) and retrieval-induced enhancement (T-B) emerged between the two groups (forgetting: *t*(22) = 3.233, *P* = 0.004, *d* = 1.324, 95% CI = [4.21, 19.27]; enhancement: *t*(22) = 2.256, *P* = 0.034, d = 0.924, 95% CI = [0.78, 18.44]), in the absence of a difference in hit rates for B items (*t*(22) = 0.824, *P* = 0.419, *d* = 0.338, 95% CI = [−6.04, 14.01]). There was no difference in terms of learning performance in the initial study phase of the T/NT experiment and a trend for higher false alarm rates during the item recognition test in PTSD patients compared to Controls (see Table 1). Taken together, PTSD patients showed deficits in controlling retrieval, whether it involves voluntary suppression or intentional remembering of episodic memories.

Memory suppression was related to symptom severity: Suppression-induced forgetting correlated negatively with re-experiencing of traumatic events on the PDS B scale and the tendency to unsuccessfully suppress unwanted thoughts in everyday life on the WBSI scale, irrespective of PTSD diagnosis (see Fig. 1c, Table 2). By contrast, retrieval-induced enhancement did neither correlate with suppression-induced forgetting nor any of the clinical measures (Table 2), suggesting a unique relationship between suppression deficits and everyday life intrusions.

**Table 2:** Spearman’s Rank Correlations between Behavioral and Clinical Measures.

	PDS B	WBSI	Enhancement
Forgetting	−0.538* [−0.745, −0.238] (0.013)	−0.559* [−0.758, −0.266] (0.013)	−0.135 [−0.458, 0.220] (0.636)
PDS B	—	0.668* [0.420, 0.823] (0.002)	−0.262 [−0.556, 0.091] (0.325)
WBSI		—	−0.059 [−0.395, 0.291] (0.784)

*n* = 24.Values represent Spearman’s rank correlation coefficients (*r*_s_, 95% CI (in brackets), and FDR adjusted *P*-values (in parentheses). *Significant correlations (*P*_adj_ < 0.05).

### MEG results

Repeated attempts to suppress sensory memory intrusions do often not alleviate PTSD symptoms but rather lead to an upholding of this condition^3^. Corresponding with these clinical observations, we expected that deficient memory suppression in the present experiment relies on a failure to downregulate MEG gamma activity as a marker of sensory long-term memory traces^7,8^ in visual processing areas and the parietal and medial temporal cortices^6,21,22^. We hypothesized that gamma activity should be reduced during NT when compared to T trials for the Control group but not for PTSD patients.

**Figure 2:**
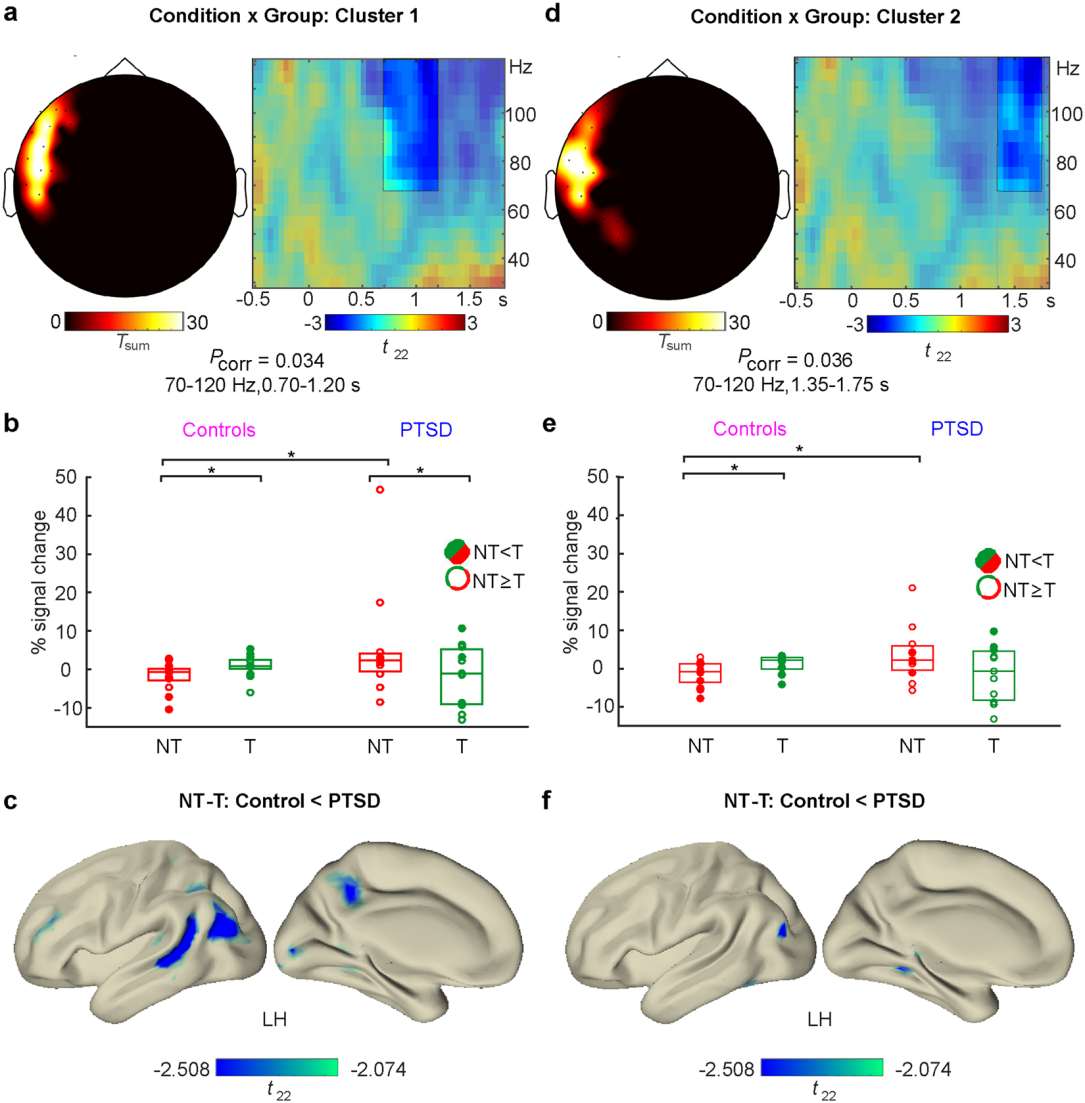
Two-way interaction between Condition (NT, T) x Group (Control, PTSD) in two significant clusters from 700 to 1200 ms (**a**–**c**) and 1350 to 1750 ms (**d**–**f**). In both clusters, results indicate reduced NT vs. T gamma power in the Control compared to the PTSD group. Top panels: Left panels in a) and d) show the sensor topography; right panels display the significant time range at the preselected frequency band (unshaded areas) averaged across sensors in negative Cluster 1 (**a**) and Cluster 2 (**d**). Middle panels: Power in the gamma frequency band at the significant sensors and in the time windows indicated by the two-way interaction effects for (**b**) Cluster 1 and (**e**) Cluster 2. Boxplots indicate median (central marks) and 25^th^ to 75^th^ percentiles (edges). Circles indicate individual participants, conforming to the NT < T hypothesis for Controls (full circles) or showing the opposite NT ≥ T pattern (empty). *Significant (*P* < 0.05) differences between conditions within and between groups as indicated by non-parametric Wilcoxon signed-rank or rank-sum tests. Bottom: Source activity of the Condition (NT-T) x Group (Control-PTSD) interaction at the time windows of sensor Cluster 1 (**c**) and Cluster 2 (**f**). Depicted *t*-values are thresholded at *P* < 0.05 (2-sided).

When compared to Controls, PTSD patients showed a deficit in reducing gamma activity in the NT vs. the T condition as evident in two temporally adjacent clusters, comprising left hemispheric sensors and spanning time windows from 700 to 1200 ms (Cluster 1: *T*_sum_ = 237.503, *P*_corr_ = 0.034; see Fig. 2a) and from 1350 to 1750 ms (Cluster 2: *T*_sum_ = 230.886, *P*_corr_ = 0.036; see Fig. 2d). Within the Control group, gamma power was reduced in the NT vs. the T condition in both clusters (Cluster 1: *Z* = 1.992, *P* = 0.046, *d* = 1.260, 95% CI = [−5.06, −0.03]; see Fig. 2b; Cluster 2: *Z* = 2.971, *P* = 0.003, *d* = 2.906, 95% CI = [−4.17, −1.25]; see Fig. 2e). This was not the case in the PTSD group, where gamma activity was even significantly increased in the NT versus the T condition in Cluster 1 (*Z* = 2.312, *P* = 0.021, *d* = 1.945, 95% CI = [0.28, 16.25]; see Fig. 2b). This paradoxical increase did not reach significance in the later occurring Cluster 2 (*Z* = 1.867, *P* = 0.062, *d* = 1.362, 95% CI = [−1.24, 10.56]; see Fig. 2e). When we compared condition-specific gamma activity between PTSD patients and Controls, we observed that these effects were due to a difference in the NT condition (Cluster 1: *U* = 37, *P* = 0.047, d = 0.894, 95% CI = [−9.22, −0.13]; Cluster 2: *U* = 35, *P* = 0.035, *d* = 0.957, 95% CI = [−9.23, −0.16]), but not in the T condition (Cluster 1: *U* = 63, *P* = 0.649, *d* = 0.202, 95% CI = [−4.36, 9.87]; Cluster 2: *U* = 64, *P* = 0.691, *d* = 0.178, 95% CI = [−2.85, 8.99]). Next, we tracked the cortical origin of these effects in the respective time windows for each cluster (700–1200 ms for Cluster 1; 1350–1750 ms for Cluster 2) via source reconstruction (see Fig. 2c and f). Source analysis revealed that source activity of the Condition × Group interaction for Cluster 1 comprised several left hemispheric areas exclusively, all of which had been previously associated with episodic retrieval of sensory information: the middle occipital gyrus, superior and inferior portions of the parietal cortex, angular gyrus and precuneus and middle/superior temporal gyrus^22^. The peak source difference was located in the left temporal gyrus (MNI coordinates: −46 −40 0). Source activity for the later Cluster 2 peaked in the left hippocampus and left parahippocampal gyrus (MNI coordinates −26 −40 0) with additional left hemispheric sources in sensory processing regions in fusiform, inferior and middle temporal and middle occipital cortex. Taken together, our data suggest that PTSD patients showed an initial increase of sensory memory traces in a temporal-parietal-occipital network followed by a sustained deficit in downregulating activity in areas related to sensory memory processing^6,23^.

**Figure 3:**
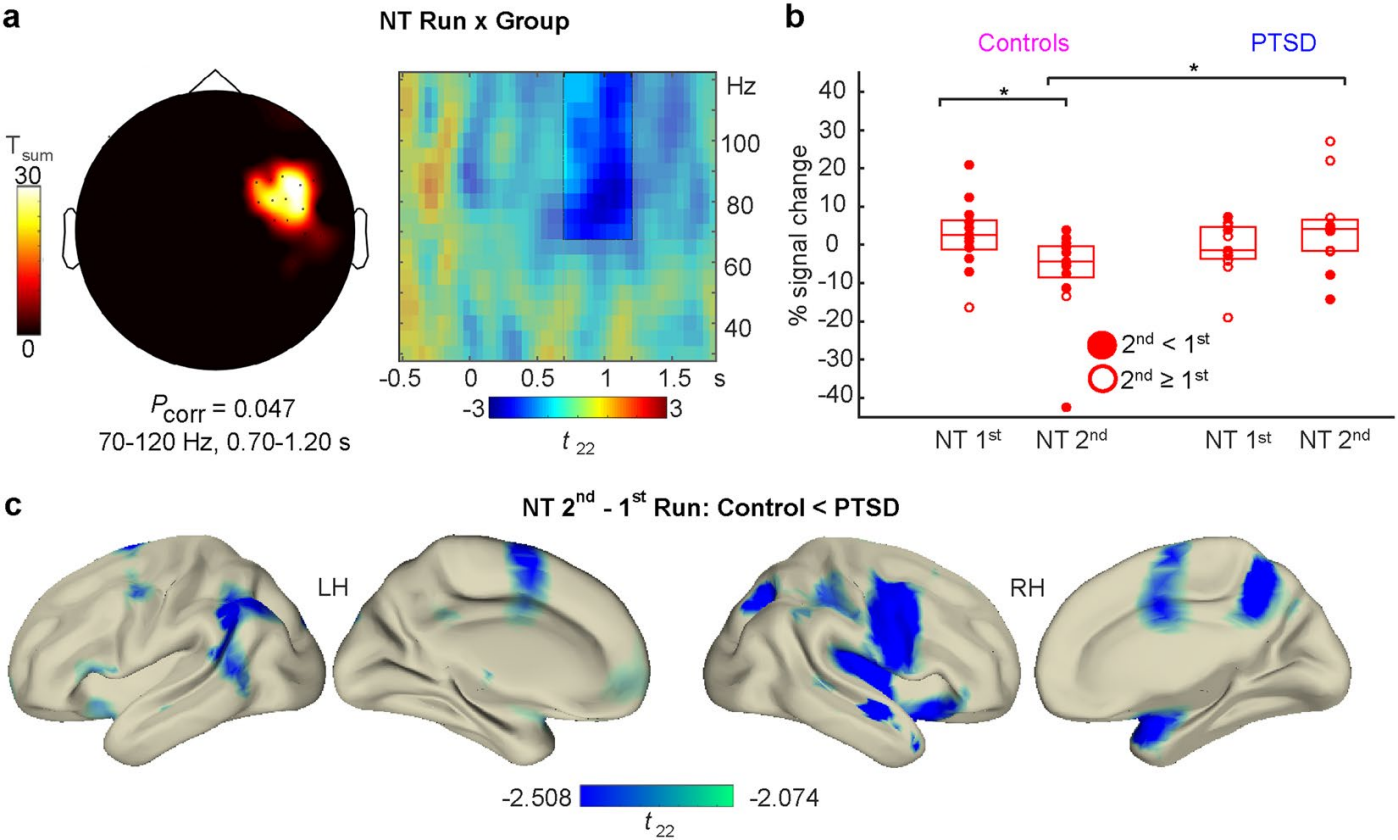
Two-way interaction between Run (2^nd^, 1^st^) x Group (Control, PTSD) for the NT condition at sensor level. (**a**) Sensor topography (left panel) and significant time range at the preselected frequency band averaged across the significant sensor cluster (unshaded area, right panel). Results suggest reduced gamma power in the second versus the first run in the Control when compared to the PTSD group. (**b**) Power in the gamma frequency band at significant sensors from 700–1200 ms as indicated by the cluster statistic. Boxplots indicate median (central marks) and 25^th^ to 75^th^ percentiles (edges). Circles indicate individual participants, conforming to the 2^nd^ < 1^st^ run hypothesis for Controls (full circles) or showing the opposite 2^nd^ ≥ 1^st^ pattern (empty). *Significant (*P* < 0.05) differences between 2^nd^ and 1^st^ run in the NT condition within and between groups as indicated by non-parametric Wilcoxon signed-rank or rank-sum tests, respectively. (**c**) Cortical sources of the Run (2^nd^ – 1^st^) × Group (Control-PTSD) interaction in the NT condition at 70–120 Hz between 700–1200 ms. Depicted *t*-values are thresholded at *P* < 0.05 (2-sided).

If gamma power indeed tracks the successful suppression of a memory trace, then it should gradually decrease from the first and to the second half of the NT runs for Controls but not for PTSD patients. Indeed, an initial 3-way Run (2^nd^, 1^st^) × Condition (NT, T) × Group (Control, PTSD) interaction analysis yielded a power modulation between the first and the second half in the gamma frequency band (500–1100 ms, *P*_corr_ = 0.013). When followed up with a Run × Group interaction in the NT condition, gamma power appeared to be decreased in the second half for the Control versus the PTSD group at right lateral sensors over the course of the NT trials between 700–1200 ms (*T*_sum_ = 224.277, *P*_corr_ = 0.047; see Fig. 3a). Within the Control group, there was a significant decrease in Run 2 versus Run 1 (*Z* = 2.900, *P* = 0.004, *d* = 2.707, 95% CI = [−16.16, −3.00]), that contrasted with a non-significant numerical increase within the PTSD group (*Z* = 1.511, *P* = 0.131, *d* = 1.024, 95% CI = [−5.12, 14.25]; see Fig. 3b). Whereas gamma power did not differ in Run 1 between groups (*U* = 54, *P* = 0.331, *d* = 0.423, 95% CI [−2.97, 8.79]) a difference emerged in Run 2, with significantly lower gamma power in the Control than in the PTSD group (*U* = 33, *P* = 0.026, *d* = 1.023, 95% CI = [−18.17, −1.06]). This interaction pattern localized to a widespread, bilateral network of reduced activity in neural generators in the parietal, occipital, insular, pre- and postcentral, temporal, cingular and frontal cortices, the right hippocampus and parahippocampal gyrus, and subcortical structures such as the amgydala, thalamus and caudate nucleus. The pattern displayed a negative peak at the right cuneus (MNI coordinates: 16 −100 30; see Fig. 3c).

## Discussion

PTSD is a disorder characterized by the recurrent and uncontrollable intrusion of traumatic memories^3^. Patients tend to try to suppress these intrusions, a strategy that proves to be highly dysfunctional and potentially aggravating re-experiencing symptoms and emotional distress. Our data provide the first biomarker for this phenomenon in a behavioral test with high ecological validity. When trying to suppress unwanted memories, PTSD patients could not forget and did not decrease their MEG gamma power in a time window (>700 ms) that is typically associated with conscious memory retrieval^17,19,23^. Interestingly, gamma power in the NT condition was higher in PTSD patients when compared to the T condition between 700 and 1200 ms. This suggests that PTSD patients are not only impaired in suppressing unwanted memories, but experience a rebound of sensory memory representations when trying to do so^3,24^.

Intriguingly, we did not observe any clear difference in the recruitment of top-down executive control mechanisms between Controls and PTSD patients in the contrast between NT and T conditions. fMRI studies consistently report higher neural activity in prefrontal cortical regions to drive inhibitory downregulation of posterior regions^18,20,25^. It is widely believed that gamma power correlates with a higher BOLD response^26^ and indicates higher neural activity in general^27^. Furthermore, frontal gamma power indicates the recruitment of top-down control networks^28^. For these reasons we would have expected inhibitory control processes to reflect in increased gamma power in the prefrontal cortex in the contrast between NT minus T conditions. However, we were not able to observe any distinct modulation of gamma activity in top-down control areas in the prefrontal cortices in the gamma band. Furthermore, previous studies have shown an increase of theta and alpha oscillatory power in the NT versus the T condition as markers of inhibitory control^23,29^. As indicated by our supplementary analyses, no such power increase occurred when comparing oscillatory power between Controls and PTSD patients (see Supplementary Data). In sum, our data only show a relative downregulation of gamma power and corresponding reduced source activity in regions that are targeted by inhibition, such as the hippocampus and visual cortical areas^6,21,25^. Some evidence for the involvement of executive control regions emerged when comparing gamma power in the NT condition between late and early runs. In contrast to Controls, PTSD patients were not only unable to downregulate neural activity with repeated suppression attempts in temporal and parietal networks engaged in storage and reactivation of unwanted memories. PTSD patients also showed less reduced gamma activity in frontal regions such as the inferior frontal gyrus and the anterior cingulate cortex, areas known to be engaged in the inhibitory control of unwanted memories^6,20,25^. This can be interpreted as indicating that PTSD patients apparently tried to suppress unwanted memories throughout the experiment, as suggested by continuous activity in inhibitory control regions, but are unsuccessful in doing so. However, the reduction of frontal gamma activity was only part of a widespread reduction of gamma power across runs in controls, with a maximum decrease again at right visual cortical locations. Thus, a clear separation of control and target areas remains difficult in the present data.

It has to be noted that our conclusions are derived from a rather small sample size. Despite the obvious limitations, the present data are unique and of utmost relevance for two reasons. First, too few studies so far have gathered empirical data on the effects of traumatic stress in refugees, which is necessary to develop effective medical strategies to deal with the immediate medical and socioeconomic challenges due to the current refugee movements^2^. Refugees and asylum seekers are often excluded from or do not seek medical treatment and if, often are not able or willing to participate in demanding cognitive neuroscience studies. In the light of these problems, it becomes evident that data such as ours are precious in understanding a rarely studied population with abundant mental health problems. Second, our sample allowed us to isolate PTSD symptomatology from the effects of depression and exposure to traumatic life events on memory. All of our participants were heavily traumatized and displayed a similar amount of depression so that patient and control groups specifically differed in terms of their diagnosis of PTSD. Typically, even in methodologically well-controlled studies, these factors are confounded. Therefore our study overcomes many interpretational problems of previous studies and presents a unique opportunity to shed light on the mechanisms underlying PTSD.

Some of our results deserve further exploration in future studies. PTSD patients did not only fail to effectively suppress unwanted memories, but they also showed deficits in the retrieval practice of desired memories. This result, though unexpected, fits with the observation that PTSD patients often show difficulties in remembering sensory and contextual details of non-traumatic episodic memories^3^. A mounting impairment in memory performance for T items with repeated retrieval attempts may point to a disruptive hyper-activation of the trauma-related memory network, which is highly accessible in PTSD patients^30^. Each retrieval attempt may eventually activate traumatic memories that, in turn, increasingly interfere with retrieval of non-traumatic episodes (see also Supplementary Data and Fig. S3). However, deficits in retrieval-induced enhancement did neither correlate with any of the clinical measures nor with MEG activity. At present, our study has to remain speculative with respect to this result.

Our results pave the way for future research. Longitudinal studies should investigate whether failures in memory suppression are a precursor or a consequence of PTSD. Furthermore, it is of high interest to show how gamma power as a marker of deficient memory suppression relates to abnormal connectivity patterns in PTSD in the same frequency range^31^, to known structural brain differences that predict susceptibility to PTSD^32^, and to genetic and epigenetic factors^10^.

Our study has important practical implications. In line with a long history of therapeutic knowledge and experimental evidence^3,24,33^, our data advise against the unwary application of memory suppression as a psychotherapeutic technique to counteract memory intrusions^5,6^: A brief training to suppress unwanted memories in severely traumatized PTSD patients does not result in an immediate reduction of unwanted memories but shows no effect. A relative increase of MEG gamma activity rather suggests that suppression attempts result in a paradoxical upregulation of sensory long-term memory traces. However, although suppression may not be an immediately effective mean to treat PTSD in all patients, our finding bears strong relevance to therapy research for several reasons. First, the success of therapeutic interventions should be reflected in the patients’ ability to modulate gamma activity in service of memory suppression. Second, being able to flexibly regulate gamma activity in the service of suppressing even neutral memories may act as a resilience factor against PTSD. Tracking this ability on the basis of the present results can aid in tailoring individualized prevention and treatment strategies.

Our data show evidence for deficient memory suppression in PTSD patients, and demonstrate that this effect is related to a dysregulation of activity in the gamma frequency band. This novel biomarker of deficient memory suppression can aid in the identification of risk and resilience factors and in developing and adapting psychotherapeutic methods. Developing strategies that can be employed effectively and economically is of utmost relevance to deal with current sociopolitical and humanitarian challenges posed by the high numbers of refugees.

## Methods

The study was approved by the Ethics Committee of the University of Konstanz, Germany, conducted in accordance with the Declaration of Helsinki (1964/2013), and written informed consent was obtained from subjects and, in case of minors, additionally by their legal guardians, prior to participation. For each participant, the study comprised a single T/NT experiment which was either preceded or followed by a structured clinical interview.

### Participants

Twenty-four refugees (5 female) from diverse European, African and Asian countries completed the full study procedure. Sample size was determined based on two previous studies with a comparable group × condition study design in a similar experimental procedure and sample. According to T/NT studies investigating similar samples and hypotheses, a total sample of size of 12 participants would be required to find a Group × Condition effect^5^, and a minimum group size of 7 would be required to find a Condition effect within group^34^, as calculated based on rather large effect sizes^5,34^. Our previous T/NT studies in healthy participants involving electrophysiological methods and a similar amount of stimulus materials as the present study, one using a similar item recognition test^19^, the other using picture cues as in in the present study^23^, suggest a sample size of 12 or 10 participants, respectively. We decided to include a total of 24 participants in order to enable full counterbalancing of conditions and stimulus materials and warrant overall comparability with previous research^17,19,23,25^. We were aware that our sample size may be quite optimistic in finding reliable effects when the total sample is divided into two subject groups based on clinical parameters. However, the extremely low availability of the participant group of interest justified this decision. Based on the results of an extensive structured clinical interview, participants were divided into two subgroups with eleven (two female) participants meeting diagnostic criteria for PTSD^9^ (see Table 1). Unequal group sizes resulted from the double-blind protocol applied in this study.

Participants received 50 € for compensation and access to subsequent psychological counseling if requested. All conversation and instruction was held unaided, if participants had sufficient language skills in German or English. In case participants were unable to engage in a natural conversation during recruitment or declared themselves unable to follow, the interview and the instructions were provided in the participants’ native tongue with the aid of interpreters. Interpreters were known to the involved clinicians as experienced translators with years of experience in translating between German and their native tongues in therapy, court and official issues. Assistance of a translator was required for ten participants of the control group and eight participants of the PTSD group. Three members of each group did not rely on assistance of translators. No significant difference between the control and the PTSD group in terms of relying on a translator emerged (*OR* = 1.25, *P* = 0.590, ϕ = 0.048, 95% CI = [0.1964, 7.9561]). All subjects had normal or corrected-to-normal vision.

Initially, 36 participants (seven female) with a mean age of 23.3 years (range 16–35) were recruited. Twelve of the participants (two female) were excluded from further analyses due to the following reasons: Malingering in the clinical interview (*n* = 1), incomplete measurements (*n* = 2), falling asleep or excessive movement during MEG data acquisition (*n* = 4), less than 50% of learned items in the Test-Feedback Recall phase of the T/NT procedure (*n* = 3), or high false alarm rates resulting in final recognition performance at chance level across conditions (*n* = 2). Malingering was indicated by obvious inconsistencies in the narrative of the flight together with an apparent gain in reporting exaggerated symptom severity. Incomplete measurement occurred due to MEG technical error. Alertness and excessive movement were determined through camera surveillance of the MEG chamber, where head movements of the subjects were monitored throughout the experiment. Poor learning and overall memory performance was determined across conditions and was applied as an exclusion factor as chance level performance possibly indicates a lack of understanding or cognitive ability to follow the experimental instructions. Five of the excluded subjects fulfilled the DSM-IV criteria for PTSD. Included and excluded subjects did not differ regarding to PTSD diagnosis (*OR* = 0.844, *P* = 0.813, ϕ = 0.040, 95% CI = [0.21, 3.43]) and clinically relevant depression (BDI-II cutoff ≥ 17; *OR* = 0.357, *P* = 0.157, ϕ = 0.239, 95% CI = [0.09, 1.49]). All exclusion criteria were set before conduction of the study and exclusion was decided before unblinding of clinical status.

### Materials

#### Clinical interview

PTSD and depression diagnoses and thought suppression were assessed by trained clinical psychologists. PTSD was diagnosed by means of the German version of the PDS, a well-established questionnaire to measure PTSD symptoms^13,14^. In case the participant was still a minor below 18 years (*n* = 2), the University of California at Los Angeles Post-traumatic Stress Disorder Reaction Index was used as the interview procedure and the data were subsequently transferred to the PDS scoring^35^. The PDS used in the present experiment shows good psychometric properties^14^ and allows for PTSD diagnosis according to DSM-IV criteria. PTSD symptom severity can be assessed by summarizing the 17 symptom items, resulting in a severity range of 0–51. In particular, we focused on the initial questions assessing number and types of experienced traumatic events (i.e., ‘trauma load’^10^) and on the sum score of the re-experiencing subscale, measuring the frequency of intrusive memories of traumatic events, corresponding to the DSM-IV criterion B. The German version of the BDI-II was used to measure depression scores^11,12^. In line with previous research, we used a cut-off value of ≥17 to identify participants suffering from a clinically relevant depression^12^. Thought suppression was assessed by means of a German translation of the WBSI^15,16^. The WBSI is comprised of several subfactors^36,37^, with high scores on the composite measure primarily reflecting unsuccessful suppression, i.e., the intrusion of unwanted thoughts despite efforts in suppressing them.

#### Stimulus material

The stimulus material used in the T/NT paradigm consisted of 54 black and white pictures of doors^38^ and 108 neutral, colored pictures of everyday objects^39^. Each participant studied 54 door-object pairs of pictures, 18 of which subsequently appeared in the T, B and NT condition, respectively. An equal number of previously unstudied everyday objects was presented in the final memory test as distractors. Stimuli were presented counterbalanced across subjects and conditions and were matched on properties known to influence memory performance (semantic category, familiarity, complexity, imaginability, and color)^39,40^.

### T/NT procedure

The T/NT task consisted of three phases (see Fig. 1a)^4^, a training phase comprising initial study and an intermediate test-feedback recall test, the T/NT phase, and a final recognition test phase. The phases were separated by breaks: Between training and T/NT there was a break of *M* = 51 min 19 s (*SD* = 09 min 50 s) used for MEG preparation and between T/NT and recognition test phase there was a break of *M* = 15 min 10 s (*SD* = 04 m 21 s). Break lengths did not differ between PTSD patients and Controls (*t*s <1.699, *P*s >0.115, *d*s <0.720).

In order to minimize burden on the patients, behavioral testing in the study and recognition phases was completed outside of the MEG chamber, in the same separate room in order to minimize confounding context effects. In the study and memory test phase, participants were seated in approximately 70 cm distance from a 20 inch monitor with a resolution of 1152 × 864 pixels. Only the T/NT phase took place inside the MEG chamber. For the duration of the T/NT phase participants laid on their back. Stimuli were presented on a ground glass screen located at appropriate viewing distance with a resolution of 800 × 600 pixels. The T/NT phase lasted for 28.5 minutes plus a self-paced break between the two runs with a duration of *M* = 6 min 13 s (*SD* = 01 min 52 s), which again did not differ between groups (*t*(22) = 1.062, *P* = 0.300, *d* = 0.424, 95% CI = [−2 min 24 s, 0 min 46 s]).

Throughout all phases of the experiment, participants were instructed to fixate on the center of the screen and to avoid eye movements to either direction. Experimenters administering the T/NT task together with MEG measurements as well as the participants themselves were blind regarding the participant’s diagnosis status resulting from the interview until completion of data collection. In all phases of the experiment, trials started with a blank black screen (inter-stimulus interval, 700 ms), followed by a fixation cross (jittered from 1000–1500 ms; 500 ms in test-feedback trials). Participants indicated their responses with their right hand in all phases of the experiment.

#### Training phase

In the training phase, participants were first instructed to silently memorize pairs of pictures, consisting of a centrally presented door (memory cue) subtending 3.7 × 5.1 cm on the screen and the picture of an everyday object presented in the lower half (visual angle: −4°) of the left or right visual field (visual angle ± 6°) subtending a maximum of 6.9 × 9.9 cm against a black background. Both stimuli were briefly shown together for 186 ms, followed by presentation of the memory cue alone for 3300 ms. Brief presentation was chosen in order to avoid saccades to laterally presented stimuli^41^. After a study block of six pairs of pictures, six corresponding test-feedback recall trials followed. In each of these trials, one of the previously studied memory cues was presented for 2000 ms accompanied by a rectangular placeholder indicating the visual field position (left or right) of the associated target. After a blank screen for 500 ms, participants were shown an array of all of the six everyday objects presented in the preceding study block and were instructed to indicate the correct target by mouse click, terminating stimulus display within 9000 ms^42^. Participants received feedback regarding their choice (correct/ wrong). In case of a wrong choice, the correct pair was shown once again in the same way as during initial study, but with the cue only shown for 1500 ms in total (not displayed in Fig. 1a). Otherwise, the next test-feedback trial followed immediately. After completion of the six test-feedback trials, participants were shown the percentage of correct answers they achieved. For the first study and test-feedback cycle, a conservative learning criterion of 100% was established in order to prevent success through random guessing, afterwards the criterion was set to 66%. If participants did not meet the learning criterion, the previously presented six pairs were re-presented in another study block, followed by corresponding test-feedback trials up to a maximum of three such repetitions. If participants were not able to reach the criterion, the experiment was continued anyway, but participants were excluded from further analysis if learning rates across all training blocks was below 50 percent (see above). In total, nine training blocks containing study and test-feedback recall on six items each were presented.

#### T/NT phase

After setting up MEG acquisition (see below), participants completed the second phase of the experiment inside the MEG chamber (see Fig. 1a). Eighteen of the previously studied cue-target pairs were assigned each to the T and NT condition. The memory cues were presented against a black background for 2000 ms in pseudo-random order with the restriction that no condition was repeated more than three times in succession. The condition (T or NT) was indicated by either a yellow or blue colored frame surrounding the centrally presented cue subtending 5.2 × 7.5 cm. Assignment of frame colors to condition was counterbalanced across participants. The T/NT phase was separated into two runs, allowing participants a break of self-paced duration after half of the trials. Each framed cue was presented six times per run (12 repetitions in total), resulting in 36 × 6 = 216 trials per run and a total of 36 × 12 = 432 trials.

Participants were instructed to selectively and covertly retrieve (T) or suppress (NT) target memories upon presentation of the associated memory cue. In the T condition, we asked subjects to actively search in memory for the target and to visualize it in as much detail as possible. For the NT condition, we stressed direct suppression instructions, requesting that participants should try to intentionally push any thought about the target memory out of consciousness without thinking of any alternative thoughts^17,18^.

#### Recognition test

Finally, retention for previously studied target items was assessed by means of an item recognition test (Fig. 1a). Participants were sequentially presented with targets from all three conditions (T, NT, B) as old items and an equal number of previously unseen new distracters. Stimuli were presented in nine consecutive blocks, each consisting of twelve randomly presented pictures. These blocks were matched regarding stimulus type (old/new), condition, and visual field of initial presentation (left/right). Participants were instructed to indicate by button press whether the presented item was studied before on a confidence rating scale from 1 (e.g., sure not to have seen the target before) to 6 (e.g., sure to have studied the target before). Response key assignment was counterbalanced across participants. Participants were instructed to respond as fast and as accurate as possible upon onset of the stimulus. Each stimulus was shown for 186 ms at the center of the screen subtending 6.9 × 9.9 cm against a black background, followed by a central fixation cross for up to 1800 ms. If participants did not indicate their decision within this time, the scale from 1 to 6 appeared centrally on the screen for up to 3000 ms, prompting an answer. After this task, we additionally asked subjects for a source judgement for all presented items they rated as old (see Supplementary Behavioral Data).

### Data analysis

#### Behavioral data analysis

Behavioral data analyses were performed using IBM SPSS Statistics, version 21 (IBM Corporation, Armonk, New York, USA) and MATLAB 2015b (The MathWorks Inc., Natick, Massachussetts, USA). To evaluate effects of the T/NT manipulation on retention, memory performance was extracted for all conditions from the final recognition test. Depending on counterbalancing condition, responses from 1 to 3 (or 4 to 6) were counted as “old” judgments and responses from 4 to 6 (1 to 3) were regarded as indicating “new” items. “Old” responses to previously studied items were regarded as hits, i.e. correct recognition responses. The first block (containing two old items from each condition and six new items) of the recognition test was considered as a practice block and omitted from further analysis. In line with previous T/NT research^19,20,23,25^, analyses were restricted to learned stimuli, as indicated by successful retrieval in the last test-feedback cycle. We conducted a two-way repeated-measures analysis of variance (ANOVA) including the factors Group (Control vs. PTSD) and Condition (T, NT, B). Significant interaction effects were followed up by planned, uncorrected, pairwise comparisons by means of two-sided *t*-tests. We further investigated differences in false alarm rates and learning rates in the test-feedback recall test between the diagnosis groups in two-sided between-group *t*-tests. For being derived from completely new items and shown randomly intermixed with items from T, NT, and B conditions during the recognition phase, false alarms could not be analyzed separately for the three experimental conditions.

*T*-values were corrected for unequal variances when indicated by significant Levene’s tests as was the case for false alarm rates. Cohen’s *d* was calculated as an effect size measure using the *t*-values. For between group analyses, *d* was corrected taking into account unequal sample size and for dependent measures for correlation between variables^43^. Nonparametric tests (Mann-Whitney-*U* and odd’s ratio *OR*) were calculated for all between-group analyses involving clinical variables, since the observed values did not show a Gaussian distribution as indicated by Kolmogorov-Smirnov tests (*Z*s <1.24, *P*s >0.05). Confidence intervals for the median differences between groups or conditions were calculated according to the Hodges-Lehmann estimator. Cohen’s *d* for non-parametric median differences were calculated as *d* = 2r/$\sqrt{1-{\rm{r}}2}$ with *r* = z/$\sqrt{{\rm{N}}}$^44^. For all reported statistical tests, significance level was set at *α* = 0.05.

We calculated Spearman’s rank correlations to determine whether suppression-induced forgetting (B-NT) was related to clinical measures of re-experiencing symptoms (PDS B) as well as thought suppression (WBSI). *P*-values from the resulting 6 correlation tests were adjusted according to the false discovery rate^45,46^ (FDR; *q* = 0.05). Confidence intervals were calculated on the basis of Fisher-Z transformed correlation coefficients (95% CI = tanh(arctanh *r*_s_ ± 0.95 * 1/$\sqrt{{\rm{n}}-3}$).

#### MEG data collection and preprocessing

Neural activity in the T/NT phase was recorded using a 148-channel whole-cortex magnetometer (MAGNES 2500 WH, 4D Neuroimaging, San Diego, California, USA) in a magnetically shielded room. Participants were placed in a supine position and instructed to lie as comfortable as possible and avoid any movement throughout the experiment. Prior to data acquisition, participant’s left and right ear canal, nasion, inion, Cz, and headshape were digitized by use of a Polhemus Fastrak® (Polhemus, Colchester, Vermont, USA). Data were continuously recorded with a sampling rate of 678.17 Hz and bandpass filtered from 0.1 to 200 Hz.

After data acquisition, neuromagnetic noise detected by reference sensors was removed from the raw data using software specialized to the MEG system at the University of Konstanz (www.pecat.eu). Continuous recordings were segmented into epochs lasting from 1500 ms before until 3500 ms after cue presentation for further analyses. Preprocessing of the trials was accomplished using the Fieldtrip^47^ open source toolbox for MATLAB 2015b (The MathWorks Inc., Natick, Massachusetts, USA).

First, an independent component analysis (ICA) was computed to correct for blinks, eye movements and cardiac artefacts. Based on an additional visual inspection of the data, data segments containing SQUID jump and movement artefacts were deleted. For data analysis on sensor level, one defect channel was interpolated using signals from the nearest-neighboring sensors in all datasets. Next, a planar gradient configuration was calculated for each magnetometer sensor, using signals from the nearest-neighboring sensors.

For time-frequency analysis, uncombined planar gradient data were submitted to a time-frequency transformation algorithm. To avoid filter artifacts at the edges of the epochs, the data were filtered in a time interval adding 500 ms of artificial data before and after the −1500 to 3500 ms epoched data. The MEG signal was convolved with Slepian multitapers in a high frequency (30–120 Hz) range, resulting in power estimates in time bins of 50 ms and frequency steps of 5 Hz. To quantify signal changes, planar gradients were combined to facilitate interpretation from sensor-level results^48^. Event-related power change was calculated in relation to a prestimulus baseline period (−500 to 0 ms). Prior to statistical testing, the data were smoothed using a Gaussian kernel spanning two time (100 ms) and two frequency bins (10 Hz)^49^. Based on previous studies^7,8,31^ and initial inspection of the data comparing the difference between NT and T conditions between groups (Control – PTSD) we investigated the modulation of gamma (70–120 Hz) power as a measure of sensory episodic memory processing affected in PTSD (see Fig. S2a).

In order to establish a link between the current results and previous studies^17,19^ we also investigated event-related fields (ERFs) in analogy to event-related potentials from EEG studies, and time-frequency changes in the theta frequency band, both indicating success in avoiding conscious memory retrieval (see Supplementary Data, Figs S1 and S2). Furthermore, we specifically looked at power modulations in the alpha (10–14 Hz) frequency band, since alpha power increases indicated the recruitment of inhibitory control networks in previous studies^29^ (see Supplementary Data).

#### MEG data analysis at sensor level

We evaluated the differential modulation of neural activity in the NT and T conditions and in the two different Runs between and within Control and PTSD groups. To deal with the multiple comparisons problem inherent to neuromagnetic data given the high number of channel x time data points, we performed non-parametric, cluster-based, random permutation tests as implemented in Fieldtrip^47,50^.

First, we calculated the condition-specific effects across all trials in a two-way interaction analysis with the factors Condition (T, NT) and Group (Controls, PTSD). Since the cluster statistics are based on *t*-tests, this was achieved by subtracting neural activity for T condition trials from activity in the NT condition. This difference was then compared between Control and PTSD groups in a two-sided independent samples comparison. Similar to previous studies focusing on event-related potentials and low frequency oscillations^17,19,23^, we expected successful memory suppression in Controls – reflected by a reduction of power in the NT vs. T condition – but not in PTSD patients. In other words, the MEG difference between the NT condition and the T condition should be more negative in Controls than in PTSD patients.

Second, we investigated whether neural activity in the two conditions was modulated across the time-course of the experiment by taking into account Run (1^st^, 2^nd^) as a third factor^51^. To this end, we subtracted the NT-T condition difference in the first run from activity in the second run and compared this two-fold difference between Groups in a two-sided independent samples comparison. Significant three-way interactions were followed-up by two-way interaction analyses, subtracting neural activity within NT and T conditions in Run 1 from Run 2 and comparing the resulting difference between Groups. We expected a significant negative cluster to emerge for the between-run comparison in the NT condition for the Control but not the PTSD group, signifying a successful reduction of memory traces in the second half of the experiment.

Technically, in the cluster statistics approach, first a two-sided samples *t*-test was computed at each sensor-time sample in each comparison. Samples showing a *P*-value below the chosen significance level *α* = 0.05 were further considered and clustered together with significant samples in adjacent time bins and at least two neighboring sensors. Thus, cluster analyses were defined data-driven across two dimensions, allowing the identification of significant temporal and spatial portions in the respective pre-selected data types. In a second step, for each of these clusters, a *t*-statistic was calculated by summing the single *t*-values within every cluster. The multiple comparisons problem was then solved using the Monte Carlo method, shuffling the data between Groups for 10000 times and calculating reference statistics for each randomization. This allows determining the probability of the observed cluster to occur in a random reference distribution. Significance level for the second-level statistic was again set at *α* = 0.05. All analyses were performed in a time window spanning from 500 ms before the onset of memory cue presentation until 1800 ms afterwards.

The interaction analyses were followed up by means of two-sided non-parametric Wilcoxon signed-rank (within groups) and rank-sum (Mann-Whitney-*U*, between groups) tests comparing data averaged over the time-range and at the sensors constituting the respective significant cluster. Again, significance level was set at *α* = 0.05. Confidence intervals for the median differences between groups or conditions were calculated according to the Hodges-Lehmann estimator.

#### MEG data analysis at source level

In order to gain a deeper understanding of the functional significance of the effects obtained at sensor level, we conducted MEG source analyses for identifying the neural generators of the interaction effects. To this end, we used a Linearly Constrained Minimum Variance (LCMV) beamforming approach^52^, localizing the artifact-corrected, non-interpolated magnetometer single trial data to individual volume conduction models. The LCMV approach constructs adaptive spatial filters to localize amplitudes of the magnetometer signals for each grid point in the entire brain.

We co-registered individual headshapes and fiducials of each participant with the FieldTrip standard MRI (MNI ICBM152 non-linear), using semi-automatic routines in the FieldTrip and the NUTMEG toolboxes^53^. Individually realigned MRIs were segmented and used as a basis for building volume conduction models necessary to accurately reconstruct potential neural generators in the LCMV source analysis. Single trial data were filtered with a band pass filter from 50–140 Hz, close to the frequency-band of interest prior to beamforming in order to avoid frequency biases in computing beamformer weights. Signals from each single trial from −1500 to 3500 ms around cue onset were localized to virtual sensors and afterwards, the same transformation algorithms were applied to source-level as to sensor-level data. Source-level data were averaged in the significant time and frequency ranges obtained from sensor-level statistics. In order to identify the neural generators driving the difference between conditions, we conducted statistics taking into account source-level signal strength as realized in Fieldtrip. Again, source activity in the T condition was subtracted from the NT condition and compared between groups (Controls – PTSD). We applied two-sided tests and did not use any cluster correction algorithm, since source analysis was conducted in order to follow-up the results already evident at sensor level. Thus, source analysis plots show *t*-values for each voxel with corresponding *P*-values exceeding the statistical threshold of *α* = 0.05 in all obtained clusters. We used the Automated Anatomical Labeling atlas^54^ implemented in Fieldtrip to label brain regions containing the maxima/minima and cerebral cortical regions that contained at least one full grid voxel (1 cm^3^) with significant *t*-values. The reported MNI coordinates for maxima/minima refer to the highest *t*-value in the biggest cluster.

## Electronic supplementary material

Supplementary Information

## Data Availability

The datasets generated and/or analyzed during the current study and all scripts used to analyze the data are available from the corresponding author on reasonable request.
