# Supplementary material for: The neural dynamics of deficient memory control in heavily traumatized refugees
Source: Sci Rep. 2018 Sep 3;8:13132. doi: 10.1038/s41598-018-31400-x (PMC6120867; doi:10.1038/s41598-018-31400-x)
Supplement: Supplementary file 1 — Supplementary Information [file 41598_2018_31400_MOESM1_ESM.pdf]

The neural dynamics of deficient memory control in heavily traumatized refugees - Supplementary Information

Gerd T. Waldhauser[1*], Martin J. Dahl[2], Martina Ruf-Leuschner[3], Veronika Müller-Bamouh[3], Maggie Schauer[3], Nikolai Axmacher[1], Thomas Elbert[3], and Simon Hanslmayr[4]

[1] Department of Neuropsychology, Institute of Cognitive Neuroscience, Ruhr University Bochum, 44801 Bochum, Germany

[2] Max Planck Institute for Human Development, 14195 Berlin, Germany

[3] Department of Psychology, University of Konstanz, 78457 Konstanz, Germany

[4] School of Psychology, University of Birmingham, Edgbaston, B15 2TT, Birmingham, United Kingdom

Supplementary Data

Supplementary behavioral data

In case a stimulus was endorsed as old in the item recognition task, a blank screen followed for 500 ms after which subjects were asked for their source rating. To this end, the letters L (left) and R (right) appeared on the respective positions on the screen for up to 3000 ms and participants had to specify on which side of the centrally presented door the target was shown during study by pressing a corresponding button within display time.

The source memory test proved unsuccessful since the obtained source memory judgements to recognition hits did not differ from 50 % chance level in all three conditions (T: M = 55.79 %, SD = 22.72, $t(23)$ = 1.248, p = 0.225, d = 0.255, 95% CI = [-3.81, 15.38]; NT: M = 54.75 %, SD = 21.92, $t(23)$ = 1.061, p = 0.300, d = 0.217, 95% CI = [-4.51, 14.00]; B: M = 51.63 %, SD = 18.32, $t(23)$ = 0.436, p = 0.667, d = 0.089, 95% CI = [-6.11, 9.37]). This was true also if we tested for this separately in both groups (Controls: ts(12) ≤ 0.739, ps ≥ 0.474, ds ≤ 0.151; PTSD: ts(10) ≤ 1.567, ps ≥ 0.148, ds ≤ 0.320). Furthermore, there was no group difference between Controls and PTSD patients in neither of the conditions (ts(22) ≤ 1.032, ps ≥ 0.313, ds ≤ 0.421). Thus, further analyses of behavioral and MEG data involving source memory performance were considered unreliable and therefore omitted. This observation is possibly due to symptoms of overgeneral episodic memory in individuals affected by trauma and depression[1], making it difficult for our participants to retrieve specific details of previous experiences.

ERFs and theta power

ERF activity for the T and NT condition was obtained by averaging time-domain signals from the artifact-corrected trials for each condition in each subject, time-locked to cue onset. Planar gradients were then combined on the individual averages to facilitate interpretation from sensor-level results. Finally, we applied a finite impulse response low-pass filter of 15 Hz and a baseline correction with a prestimulus time window from 200 ms to 0 ms prior to statistical testing. For source level analyses, a low-pass filter was applied at 15 Hz prior to beamforming. For time-frequency analyses of theta activity, a time-frequency representation from 2-30 Hz was derived from the artifact-corrected single-trial data by means of Morlet wavelets (width 5), resulting in oscillatory power in time bins of 50 ms and frequency steps of 1 Hz. For source level analysis, single trials were filtered with a low pass filter at 8 Hz prior to beamforming (see Fig. S2 for results).

Analyses of ERFs yielded a differential modulation of a late parieto-occipital ERF component between 914 and 1104 ms, interacting between Group x Condition: T_{sum} = 5698.868, p_{corr} = 0.028, Fig. S1a). For theta oscillatory power, an initial two-way interaction analysis was only marginally significant at a right central cluster from 450 to 1650 ms (T_{sum} = 479.250, p_{corr} = 0.086; see Fig. S2a).

In order to test whether these results replicate our previous findings in healthy students[2,3], we followed up on these effects. ERFs and theta power for the NT condition were reduced when compared to the T condition in the Control group (ERF: Z = 2.970, p = 0.003, d = 2.906, 95% CI = [-469.67, -126.05], Fig. S1b; theta: Z = 2.411, p = 0.016, d = 1.131, 95% CI = [-16.14, -2.59], Fig. S2c). This was not the case in the PTSD group (ERF: Z = 0.800, p = 0.424, d = 0.497, 95% CI = [-110.13, 319.78]; theta: Z = 1.245, p = 0.213, d = 0.526, 95% CI = [-1.778, -9.518]). However, ERFs differed between PTSD patients and Controls in the T (ERF: U = 35.00, p = 0.035, d = 0.957, 95% CI = [32.60, 733.59]; U = 28.00, p = 0.011, d = 1.200, 95% CI = [4.40, 25.63]) but not in the NT condition (ERF: U = 61.00, p = 0.569, d = 0.250, 95% CI = [-441.36, 237.33]; theta: U = 67.00, p = 0.82, d = 0.107, 95% CI = [-7.49, 11.17]).

Neural source activity for the ERF interaction pattern emerged with a peak in the left middle occipital/ angular gyrus (MNI coordinates: -34, -60, 20) and in the left angular, right fusiform, bilateral lingual, and the left middle occipital and temporal cortices (see Fig. S1c). This suggests that the ERFs indicate the modulation of episodic retrieval including processing of reactivated visual sensory memory traces[4]. The peak theta power interaction difference was observed in the left postcentral/ left inferior parietal gyrus (MNI coordinates: -46, -30, 50) and additional sources in the left midtemporal cortex (see Fig. S2d).

For ERFs, a three-way interaction effect involving the factors Run x Condition x Group in a time window from 1352 to 1498 ms only trended to be significant (T_{sum} = 3414.275, p_{corr} = 0.052; Fig. S1d). This indicates a relative increase of magnetic field strength for the NT condition versus a decrease in the T condition in the Control group compared to a reversed effect in the PTSD group. Two-way Run x Group follow-up analyses within conditions did not further qualify this observation for NT ($T_{sum} \leq 5698.867$; $ps_{corr} \geq 0.124$) and T ($T_{sum} \leq 732.071$, $ps_{corr} \geq 0.558$). For theta power, 3-way Run (2nd, 1st) x Condition (NT, T) x Group (Control, PTSD) interaction did not yield any significant clusters ($T_{sum} \leq 205.888$, $ps_{corr} \geq 0.188$)[3].

Gamma power in the T condition

We also tested for a modulation of gamma power with repeated attempts to retrieve the desired memory representation in the T condition. We obtained a non-significant positive cluster at left-hemispheric sensors spanning from 950 to 1200 ms in a two-way interaction analysis with the factors Run x Group within the T condition (T_{sum} = 131.672, P_{corr} = 0.059; see Supplementary Data and Fig. S3).

In order to get an idea about how to explain lower memory performance in the T condition in PTSD patients we also followed up on the non-significant two-way interaction effect in the T condition across runs (see Fig. S3a). Gamma power in the T condition increased in the Control group in Run 2 when compared to Run 1 (Z = 2.691, p = 0.007, d = 2.234, 95% CI = [1.15, 5.70], see Fig. S3b), presumably signifying the more successful retrieval of sensory memory traces with practice[5]. The opposite pattern emerged in the PTSD group, with gamma power decreasing in Run 2 when compared to Run 1 (Z = 2.223, p = 0.026, d = 1.806, 95% CI = [-12.63, -1.15]). Repeated retrieval in PTSD possibly renders episodic memories less rich in sensory and contextual details. This is in line with the observation that PTSD affects the retrieval of details of desired memories[1,6,7]. No difference emerged between groups when comparing gamma power within the same runs (Run 1: U = 49.00, p = 0.207, d = 0.552, 95% CI = [-8.34, 2.86]; Run 2: U = 50.00, p = 0.228, d = 0.526, 95% CI = [-2.97, 12.32]).

The most pronounced difference in source activity was observed in the left precentral cortex (MNI coordinates: -54, 0, 20). In addition, we observed widespread significant clusters in the left hemisphere spanning from the frontal gyri to the post-central gyrus, the insula, the middle cingulum and occipital, parietal, and inferior, middle and superior temporal areas that are involved in memory retrieval and visual processing (see Fig. S3c).

Effects in the alpha frequency band

To even more specifically follow up on previous studies, in particular with respect to the neural markers of inhibitory control, we calculated Group x Condition interaction effects for the alpha (10-14 Hz) frequency band[3,8]. No significant effects emerged in this analysis (T_{sum} < 28.2978, P_{corr} > 0.7322) and the same was true for a three-way interaction between Run x Group x Condition (T_{sum} < 201.503, P_{corr} > 0.1873)

Supplementary References

1. Graham, B., Herlihy, J. & Brewin, C. Overgeneral memory in asylum seekers and refugees. *J. Behav. Ther. Exp. Psych.* **45**, 375–80 (2014).
2. Waldhauser, G. T., Lindgren, M. & Johansson, M. Intentional suppression can lead to a reduction of memory strength: Behavioral and electrophysiological findings. *Front. Psychol.* **3,** 401 (2012).
3. Waldhauser, G. T., Bäuml, K.-H. T. & Hanslmayr, S. Brain oscillations mediate successful suppression of unwanted memories. *Cereb. Cortex* **25,** 4180–4190 (2015).
4. Hall, S. *et al.* The neural basis of involuntary episodic memories. *J. Cogn. Neurosci.* **26,** 2385–2399 (2014).
5. Osipova, D. *et al.* Theta and gamma oscillations predict encoding and retrieval of declarative memory. *J. Neurosci.* **26,** 7523–7531 (2006).
6. Brewin, C. R. The nature and significance of memory disturbance in posttraumatic stress disorder. *Clin. Psychol.* **7,** 203–227 (2011).
7. Schönfeld, S., Ehlers, A., Böllinghaus, I. & Rief, W. Overgeneral memory and suppression of trauma memories in post-traumatic stress disorder. *Memory* **15**, 339–52 (2007).
8. Depue, B. *et al.* ERPs and neural oscillations during volitional suppression of memory retrieval. *J. Cogn. Neurosci.* **25,** 1–10 (2013).

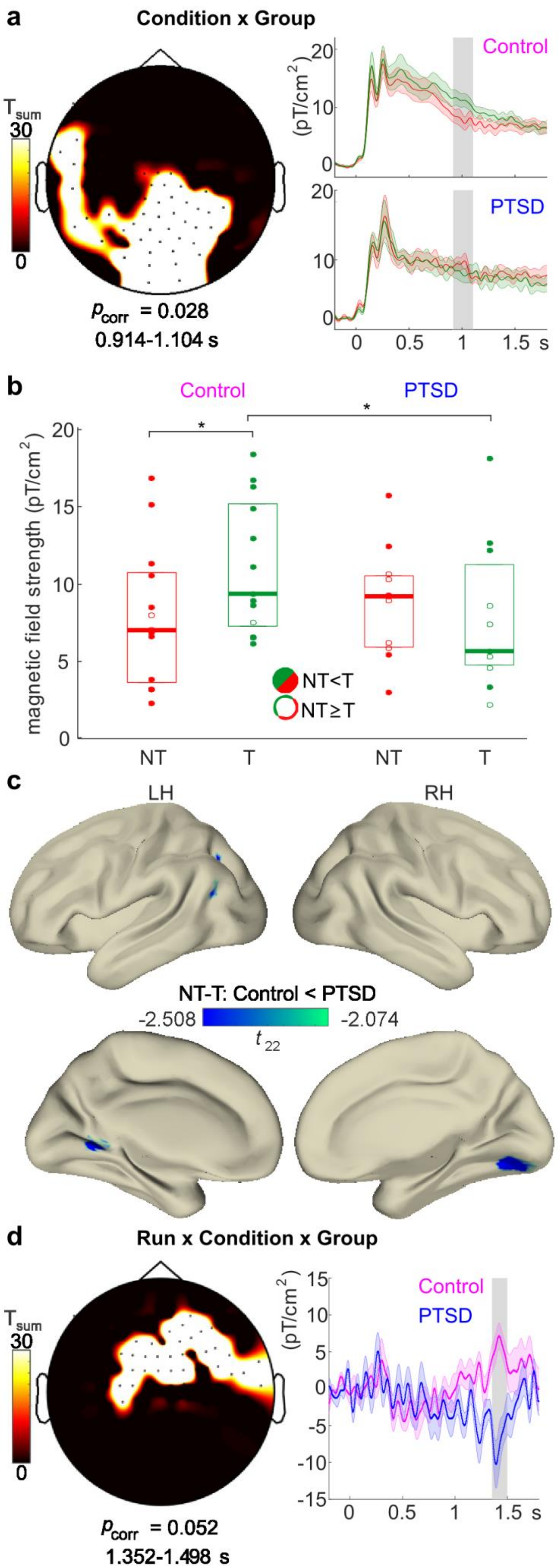
a
Condition x Group
T_{sum}
30
0
p_{corr} = 0.028
0.914-1.104 s
(pT/cm^2)
Control
PTSD
0 0.5 1 1.5 s
b
Control
PTSD
magnetic field strength (pT/cm^2)
NT<T
NT≥T
NT
T
NT
T
c
LH
RH
NT-T: Control < PTSD
-2.508
-2.074
t_{22}
d
Run x Condition x Group
T_{sum}
30
0
p_{corr} = 0.052
1.352-1.498 s
Control
PTSD
(pT/cm^2)

Figure S1. Differential modulation of ERFs depending on Condition and Run between Groups. **a)** Two-way interaction Condition x Group cluster statistics. Left panel: Topography of the significant cluster. Right panel: Grand average ERF time course together with SEM at the significant sensor cluster. Significant time range is indicated by grey shaded areas. **b)** Magnetic field strength at significant cluster. Boxplots indicate median (central marks) and 25th to75^{th} percentiles (edges). Circles indicate individual participants, conforming to the NT<T hypothesis for Controls (full circles) or not (empty). *Significant ($P < 0.05$) differences between conditions and groups. **c)** Cortical sources of the Condition (NT-T) and Group (Control-PTSD) ERF interaction from 914-1104 ms. Depicted t-values are thresholded at $P < 0.05$ (2-sided). **d)** Three-way interaction Run x Condition x Group at sensor level. Topography of the significant positive cluster between 1352-1498 ms (left panel) and grand average ERF NT-T difference wave (with SEM) in the second versus the first run in the Control and PTSD groups (right panel). The significant time range is marked by grey shaded area.

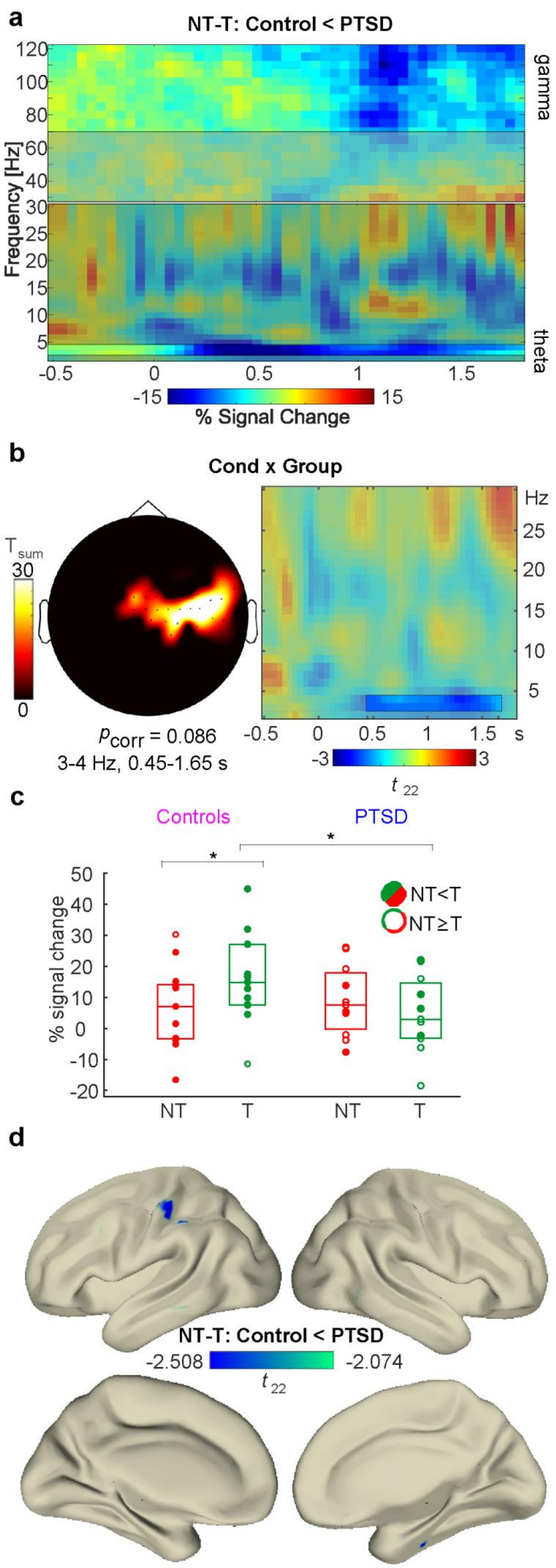
a
NT-T: Control < PTSD
120
100
80
60
40
30
25
20
15
10
5
Frequency [Hz]
gamma
theta
-0.5
0
0.5
1
1.5
-15
15
% Signal Change
b
Cond x Group
T sum
30
0
p_{corr} = 0.086
3-4 Hz, 0.45-1.65 s
Hz
25
20
15
10
5
-0.5
0
0.5
1
1.5
s
-3
3
t_{22}
c
Controls
PTSD
*
*
50
40
30
20
10
0
-10
-20
% signal change
NT<T
NT≥T
NT
T
NT
T
d
NT-T: Control < PTSD
-2.508
-2.074
t_{22}

Figure S2. a) Frequency selection based on unsmoothed data across the full time and frequency range comparing NT and T differences between Control and PTSD groups averaged across all 148 sensors in a high (30-120 Hz) and low (2-30 Hz) frequency range. Initial inspection revealed most sensitive modulation at 70-120 Hz and 3-4 Hz (unshaded areas). **b)** Two-way interaction of theta power (3-4 Hz) between Condition x Group, showing the sensor topography (left panel) of the negative cluster and the time range at the preselected frequency band averaged across the sensor cluster (unshaded area, right panel). **c)** Power in the theta frequency band at the non-significant sensor cluster. Boxplots indicate median (central marks) and 25th to75th percentiles (edges). Circles indicate individual participants, conforming to the NT<T hypothesis (full circles) or showing the opposite pattern (empty). *Significant (P < 0.05) differences between conditions within and between groups. **d)** Cortical sources of the Condition x Group interaction in the 450-1650 ms time range at 3-4 Hz. Depicted t-values are thresholded at P < 0.05 (2-sided).

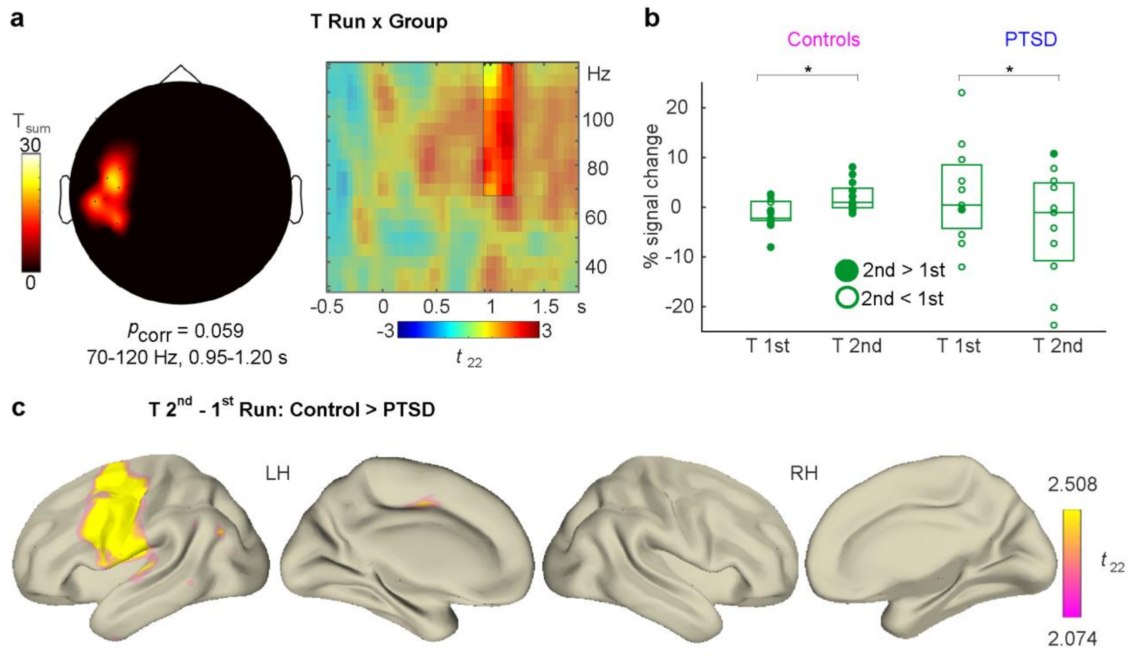

Figure S3. Differential modulation of gamma power between Run x Group within the T condition. **a)** Sensor topography (left panel) and time range (unshaded areas, right panel) for the marginally significant cluster. **b)** Power in the gamma frequency band at cluster sensors. Boxplots indicate median (central marks) and 25th to75th percentiles (edges). Circles indicate individual participants, conforming to the 2nd > 1st run hypothesis for Controls (full circles) or showing the opposite pattern (empty). *Significant ($P < 0.05$) differences between conditions within and between groups. **c)** Cortical sources of the Run x Group interaction in the 950-1200 ms time range at 70-120 Hz. Depicted *t*-values are thresholded at $P < 0.05$ (2-sided).
